# Still Temporizing with the Blank Cartridge Pistol

**Published:** 1910-01

**Authors:** 


### Still Temporizing With the Blank Cartridge Pistol.

Blank cartridge wounds cause more deaths in the annual celebration of the Fourth of July than all other factors combined. In seven years 794 deaths have been caused by this one factor! Most of the victims were bright active boys, aged from 6 to 18 years, and they were doomed to die the most awful death known to medical science, a death the agony of which is probably not paralleled even by the tortures of the Inquisition. If this annual sacrifice were really necessary, it would be far more merciful to pick out the hundred or more youths each year and deliberately shoot them. But this annual outrage is not necessary; it is entirely preventable, and the prevention rests with our city governments.

For seven years the *Journal* has heralded these facts to the world. They have been given even wider publicity by some of our public-spirited newspapers. During the past two or three years the public press generall has taken up the cry, and now no one can plead ignorance of the awful facts. Nevertheless, the average city government still views with the blind eye of callous indifference the fatalities for which it is morally responsible. Some cities have, indeed, passed prohibitory ordinances, but have not enforced them vigorously. To permit the use of blank cartridges and blank cartridge pistols in the celebration of the Fourth of July is absolutely criminal. The city council which fails to pass and to enforce ordinances prohibiting the use of these instruments of torture will hereafter be open to the charge of criminal negligence. The plea of ignorance is no longer good; to temporize further regarding the sale and use of blank cartridges and blank cartridge pistols is to aid and abet torture and murder.

These are strong words; but to use mild ones would be to trifle with the anguish and horror in scores of homes—a horror and anguish but faintly reflected in the appalling statistics on the subject. The only effective way to deal with the murderous blank cartridge pistol is to pass and to enforce an absolutely prohibitive ordinance in each city. No more temporizing should be tolerated. The agonizing deaths from this cause should cease.—*Journal A. M. A.*

